# Endoscopic electrocauterization for management of chronic perineal sinus following abdominoperineal resection

**DOI:** 10.1093/jscr/rjac194

**Published:** 2022-05-17

**Authors:** Brandon Larson, Shayan Azizi, Truong Ma

**Affiliations:** Department of Colon and Rectal Surgery, Summa Health System, Akron, OH, USA; Department of Colon and Rectal Surgery, Summa Health System, Akron, OH, USA; Department of Colon and Rectal Surgery, Summa Health System, Akron, OH, USA

## Abstract

We present a case of a chronic perineal sinus following abdominoperineal resection with management via endoscopic electrocauterization. This patient presented with 1 year of bloody, mucus drainage from a perineal wound following abdominoperineal resection for anastomotic leak and stricture from a remote low anterior resection for T2N1 rectal cancer. We describe a novel use of endoscopic electrocautery to debride, de-epithelialize and ultimately eliminate the sinus cavity. The patient’s postoperative course was uncomplicated and reported decreased drainage at 2- and 4-week postoperative follow-up. Long-term plans include sequential drain downsizing to facilitate cavity closure. Our findings suggest that endoscopic electrocauterization can safely and effectively reduce chronic perineal sinus drainage to facilitate cavity elimination, while avoiding morbidity associated with more invasive operative interventions.

## CASE DESCRIPTION

**Figure 1:**
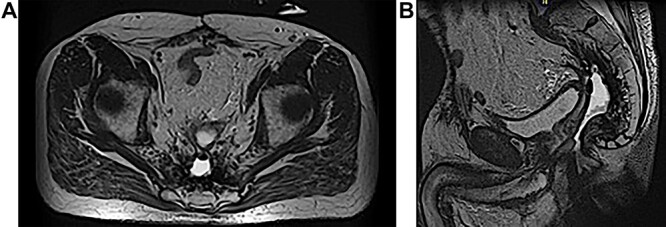
(**A** and **B**) MRI abdomen and pelvis demonstrating 2–3 cm pelvic sinus cavity with connecting tract to the perineum.

**Figure 2:**
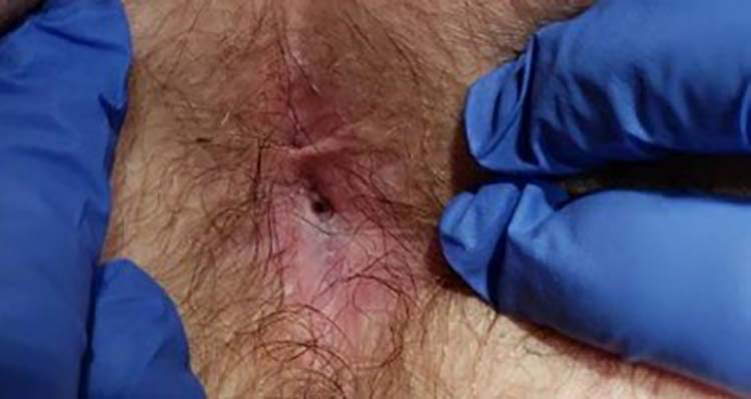
Physical exam in office showing perineal wound with sub-centimeter cutaneous opening.

A 63-year-old male presented to the Colorectal Surgery Clinic complaining of constant sanguineous, mucous drainage from a chronic perineal sinus tract. He has a history of T2N1 rectal cancer status post neoadjuvant chemoradiation followed by low anterior resection 17 years prior to presentation complicated by a chronic anastomotic leak. Management of the anastomotic leak required a transverse loop colostomy with subsequent takedown and creation of a diverting loop ileostomy. One year prior to presentation, he underwent exploratory laparotomy with total proctocolectomy and abdominoperineal resection for simultaneous distal loop ileostomy stricture and a stricture at the low anterior resection anastomosis with chronic anastomotic leak, rectal bleeding and mucus drainage. Biopsies were taken at that time given concern for pelvic recurrence which showed benign lymph nodes, fat necrosis and abscess. Since surgery, the patient reported bleeding from a perineal wound and constant passage of mucus. Magnetic resonance imaging (MRI) 1 month prior to clinic presentation showed a retained 2–3 cm sinus cavity in the pelvis with a connecting tract to the perineum (Fig. 1A and B). Exam in clinic revealed a perineal wound with a sub-centimeter cutaneous opening and scant mucous drainage (Fig. 2). Given the MRI findings and significant lifestyle disturbances caused by the chronic drainage, he was scheduled for an exam under anesthesia with endoscopic cauterization of the sinus cavity lining in hopes of obliterating the cavity epithelium.

## OPERATIVE DESCRIPTION

**Figure 3:**
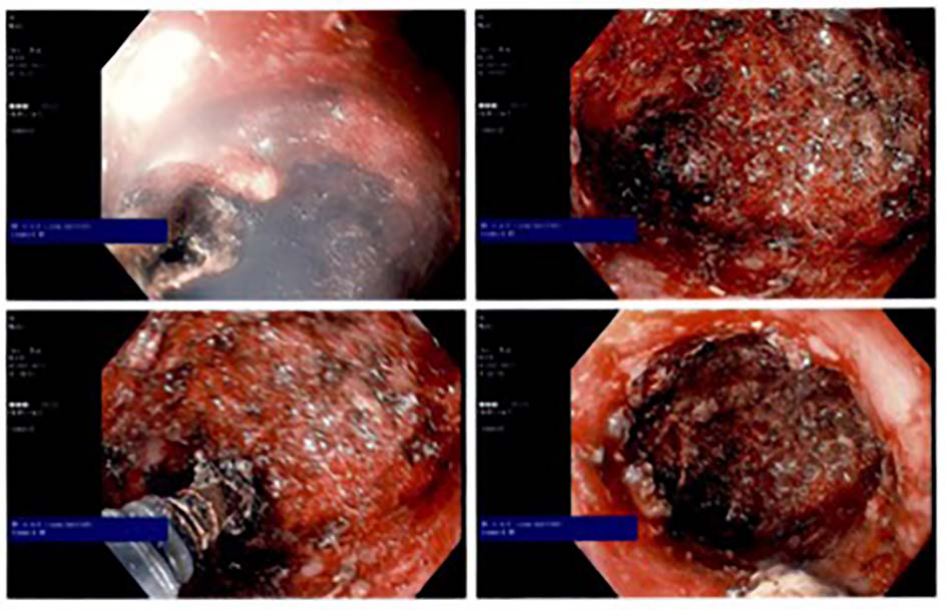
Intraoperative endoscopic photographs showing cauterized cavity epithelium.

The patient was placed in the prone jackknife position and a digital exam was performed by probing the punctate perineal wound with noted purulent drainage. The area was digitally dilated up to an area of apparent stenosis 3–4 cm into the tract which, using a probe, was noted to track up about 12 cm proximally. An esophagogastroduodenoscopy scope was then passed through the opening to view the cavity, which was roughly 3 cm in diameter and 12 cm in length. Using hot biopsy forceps and hot snare, the inner lining of the cavity was debrided with electrocautery (Fig. 3). All cavity surfaces were cauterized as able including the neck of the cavity. The cavity was then irrigated with hydrogen peroxide and a 30-French Malecot drain was placed in the opening and secured to the skin with a 2-0 silk suture.

The patient’s postoperative course was uncomplicated and he was discharged home the same day. He was seen at a 2-week postoperative clinic appointment where he endorsed decreased quantity of yellow mucus drainage from the sinus compared to preoperatively. At 4 weeks postoperative, he noted the drainage output had stabilized, although again decreased from the preoperative quantity. He is planned for repeat computed tomography imaging with rectal contrast through the Malecot drain to evaluate reduction in cavity size with planned progressive drain downsizing to allow for cavity closure.

## DISCUSSION

Chronic perineal sinus is a well-known complication of abdominoperineal resection that exists as a nonhealing wound at least 6 months after surgery. Previous studies have reported that up to 30% of patients undergoing abdominoperineal resection will suffer a chronic perineal sinus [1, 2]. Early intervention is recommended as conservative management does not typically facilitate complete closure of the sinus. Surgical management includes curettage, primary closure, wide-local excision, marsupialization and vacuum-assisted closure [3, 4]. More invasive reconstructive measures include using omental, cutaneous or myocutaneous donor sites [5–7]. Wide excision and reconstructive methods often have increased morbidity, given the additional risk of healing complications of incision and donor sites [6].

The use of sinusoscopy to visualize and debride chronic perineal sinus has been described in two existing case reports looking at four patients in total [3, 8]. Compared to these cases which utilize mechanical debridement via biopsy grasper, we introduce a novel technique of electrocauterization to de-epithelialize the sinus cavity. All patients described had a history of rectal cancer with abdominoperineal resection. Both cases had similar protocols using a slim endoscope but differed in the use of hydrogen peroxide versus saline alone for cavity irrigation. One of four patients received parenteral antibiotics for 48 hours after endoscopy due to clinical evidence of sepsis and insufflated air into the peritoneal cavity [8]. There were no other reported complications. Three patients had total follow-up times of 18–36 months and none experienced recurrence of the chronic sinus [4].

Limitations of the existing literature for endoscopic management of chronic perineal sinus include the anecdotal nature of case reports and small sample sizes. There is a need for prospective trials utilizing larger sample sizes comparing recurrence and complications of endoscopic electrocauterization to conventional methods of chronic sinus closure. While safe and replicable, potential complications of the procedure include sepsis and insufflation of air into the peritoneal cavity. Previous authors have suggested prophylactic antibiotics and a 70-day delay period for cavity maturation prior to sinusoscopy [8]. Our endoscopic technique provides the ability to visualize all aspects of the sinus cavity and provide therapeutic intervention for cavity closure without the morbidity associated with a wide excision.

## CONCLUSION

Chronic perineal sinus is a common complication of abdominoperineal resection that is often challenging to manage. Previous case reports describe simple mechanical debridement of the cavity. We report endoscopic electrocauterization for the evaluation and treatment of a chronic perineal sinus cavity. Our experience shows that this is a safe and effective technique for debridement, while avoiding the morbidity of wide excision. We propose this for initial management of a chronic perineal sinus before more invasive measures are undertaken.

## CONFLICT OF INTEREST STATEMENT

There are no conflicts of interest.

## FUNDING

No funding was received.

## References

[1] Chau A, Prodeau M, Sarter H, et al. Persistent perineal sinus after abdominoperineal resection. Langenbecks Arch Surg 2017;402:1063–9. 28840372 10.1007/s00423-017-1619-0

[2] Lohsiriwat V . Persistent perineal sinus: incidence, pathogenesis, risk factors, and management. Surg Today 2009;39:189–93. 19280276 10.1007/s00595-008-3846-z

[3] Ko S . Sinoscopic treatment of persistent perineal sinus. Tech Coloproctol 2017;21:987–9. 29147827 10.1007/s10151-017-1720-y

[4] Schaffzin D, Douglas J, Stahl T, Smith L. Vacuum-assisted closure of complex perineal wounds. Dis Colon Rectum 2004;47:1745–8. 15540310 10.1007/s10350-004-0633-9

[5] Yamamoto T, Mylonakis E, Keighley M. Omentoplasty for persistent perineal sinus after proctectomy for Crohn’s disease. Am J Surg 2001;181:265–7. 11376583 10.1016/s0002-9610(01)00561-x

[6] Mahmoud N, Kamrava A. Prevention and management of nonhealing perineal wounds. Clin Colon Rectal Surg 2013;26:106–11. 24436658 10.1055/s-0033-1348049 PMC3709982

[7] Wilson T, Welbourn H, Stanley P, Hartley J. The success of rectus and gracilis muscle flaps in the treatment of chronic pelvic sepsis and persistent perineal sinus: a systematic review. Colorectal Dis 2014;16:751–9. 24831668 10.1111/codi.12663

[8] Al-sheikh M, Cuming T, Ashraf N, Hepworth C. Sinoscopy: endoscopic washout of perineal sinus after abdominoperineal excision of the rectum. Tech Coloproctol 2015;19:431–3. 25975972 10.1007/s10151-015-1313-6

